# Evidence for inter-specific recombination among the mitochondrial genomes of *Fusarium* species in the *Gibberella fujikuroi* complex

**DOI:** 10.1186/1471-2164-14-605

**Published:** 2013-09-08

**Authors:** Gerda Fourie, Nicolaas A van der Merwe, Brenda D Wingfield, Mesfin Bogale, Bettina Tudzynski, Michael J Wingfield, Emma T Steenkamp

**Affiliations:** 1 Department of Microbiology and Plant Pathology, Forestry and Agricultural Biotechnology Institute (FABI), University of Pretoria, Pretoria, South Africa; 2 Department of Genetics, Forestry and Agricultural Biotechnology Institute (FABI), University of Pretoria, Pretoria, South Africa; 3 University of Munster, Germany

**Keywords:** Synteny, Group I intron, LAGLIDADG and GIY-YIG homing endonucleases, Incongruent phylogenies

## Abstract

**Background:**

The availability of mitochondrial genomes has allowed for the resolution of numerous questions regarding the evolutionary history of fungi and other eukaryotes. In the *Gibberella fujikuroi* species complex, the exact relationships among the so-called “African”, “Asian” and “American” Clades remain largely unresolved, irrespective of the markers employed. In this study, we considered the feasibility of using mitochondrial genes to infer the phylogenetic relationships among *Fusarium* species in this complex. The mitochondrial genomes of representatives of the three Clades (*Fusarium circinatum*, *F. verticillioides* and *F. fujikuroi*) were characterized and we determined whether or not the mitochondrial genomes of these fungi have value in resolving the higher level evolutionary relationships in the complex.

**Results:**

Overall, the mitochondrial genomes of the three species displayed a high degree of synteny, with all the genes (protein coding genes, unique ORFs, ribosomal RNA and tRNA genes) in identical order and orientation, as well as introns that share similar positions within genes. The intergenic regions and introns generally contributed significantly to the size differences and diversity observed among these genomes. Phylogenetic analysis of the concatenated protein-coding dataset separated members of the *Gibberella fujikuroi* complex from other *Fusarium* species and suggested that *F. fujikuroi* (“Asian” Clade) is basal in the complex. However, individual mitochondrial gene trees were largely incongruent with one another and with the concatenated gene tree, because six distinct phylogenetic trees were recovered from the various single gene datasets.

**Conclusion:**

The mitochondrial genomes of *Fusarium* species in the *Gibberella fujikuroi* complex are remarkably similar to those of the previously characterized *Fusarium* species and Sordariomycetes. Despite apparently representing a single replicative unit, all of the genes encoded on the mitochondrial genomes of these fungi do not share the same evolutionary history. This incongruence could be due to biased selection on some genes or recombination among mitochondrial genomes. The results thus suggest that the use of individual mitochondrial genes for phylogenetic inference could mask the true relationships between species in this complex.

## Background

The origin of mitochondria can be traced to about one billion years ago when an endosymbiotic association emerged between α-proteobacteria and the “proto eukaryotic” host cell [1,2]. Since then, the genome size of this enslaved bacterium or organelle has reduced substantially [2-4]. This reduction was mainly through the loss of genes needed for living freely, or through migration to the nucleus of genes required for functional integrity of the mitochondrion [5]. However, the processes involved in mitochondrial (mt) genome evolution differ considerably across the eukaryotic tree of life. This is strikingly evident from the diversity that eukaryotes display in terms of mt gene content, gene and genome organization, genome size and the presence of mobile genetic elements [6,7].

The mt genome is considered to be an ideal region to study eukaryotic evolution [2]. This is not only linked to its ancestral origins, but also because of its accelerated rate of evolution, which is associated with a high copy number that allows mutations to occur without lethal impact [6]. In addition, gene loss appears to be irreversible [2] and transfer of genetic material between or into mt genomes is thought to be limited [6] although transfer of genetic material between mitochondrial genomes via intron homing and/or plasmids can occur [8,9]. The mt genome is also relatively small and can thus be studied in its entirety. It is thus not surprising that mt genomes have been used in various phylogenetic and comparative studies to resolve and/or determine evolutionary relationships between or among eukaryotes at all taxonomic levels e.g [10,11]. In the fungi, partial or whole mt genome sequence data have been used to resolve relationships among various members of the Basidiomycota [12-14], and in the Ascomycota among classes such as Schizosaccharomycetes [15], Dothideomycetes [16], Eurotiomycetes [17], as well as Sordariomycetes [18-22].

In this study, we considered the feasibility of using mitochondrion-encoded gene sequences to infer phylogenetic relationships among *Fusarium* species in the *Gibberella fujikuroi* complex (GFC). The GFC represents a monophyletic assemblage of *Fusarium* species, the majority of which are of considerable agricultural, medical and veterinary importance [23]. Based on nuclear DNA sequence information and a number of phenotypic markers, this fungal complex has been separated into at least 50 species [23,24]. The use of these markers, particularly DNA sequences, has also revealed that the overall GFC phylogeny is characterized by two deep divergences that divide the complex into three large clades. These have been designated the “African”, “American” and “Asian” Clades based on the origin of the hosts or substrates from which the members of the GFC were isolated [25,26]. The exact relationships among these three clades remain largely unresolved, because phylogenies from different studies are often incongruent. For example, a multi-gene phylogeny based on beta-tubulin and calmodulin gene sequence data suggests that the “African” Clade is ancestral in the species complex [27]. However, similar reports have also been published for the “Asian” Clade based on beta-tubulin, translation elongation factor 1-alpha and histone 3 data [28,29] and for the “American” Clade based on beta-tubulin and translation elongation factor 1-alpha sequence data [30].

The causes of incongruence among and between gene trees and species trees may be ascribed to various factors, which have been extensively reviewed previously e.g., [31-33]. Although such conflicts may hold valuable clues regarding the life histories of the taxa under investigation [34], problems associated with phylogenetic inconsistencies can usually be circumvented by using multiple and information-rich DNA sequences [35,36]. Among the nuclear genes commonly employed to study *Fusarium* species in the GFC, most are highly conserved and only those encoding translation elongation factor 1-alpha and beta-tubulin are usually sufficiently variable to discriminate closely related species [25,26,37]. Any improvements in the GFC phylogeny are thus dependent on the identification and use of additional genomic regions with evolutionary trajectories matching those of the complex itself.

Few studies have previously exploited the use of mt gene sequences for solving phylogenetic questions in *Fusarium* and these are generally limited to the mt-encoded small ribosomal subunit gene *rns*[25,27]. In fact, the fully annotated mt genome for only one member of the GFC, *Fusarium verticillioides* (Saccardo) Nirenberg, is currently available in the public domain [22]. Because of the limited information available on the structure and evolution of mt DNAs in the GFC, the first objective of this study was, therefore, to fully characterize the mt genomes for a representative set of species. For this purpose, three species in the complex were used: *Fusarium circinatum* Nirenberg and O’Donnell emend. Britz, Coutinho, Wingfield and Marasas, which is the causal agent of pine pitch canker [38] and representative of the “American” Clade; *Fusarium fujikuroi* Nirenberg, which is the causal agent of Bakanae disease of rice [39] and representative of the “Asian” Clade; and *F. verticillioides* that causes seed, root, stalk and ear rot of maize [40] and that is representative of the “African” Clade [22]. To achieve our first objective, a comparative genomic approach was used to predict and annotate genes and repeat elements in the GFC species, which allowed subsequent comparisons with those described previously within *Fusarium*[19,22,41] and the Sordariomycetes [18,20,21]. The second objective of this study was to determine whether the mt genomes of these fungi have any value in resolving the higher-level evolutionary relationships in the GFC. To this end, we compared the phylogenetic trees recovered from the single gene sequences, as well as from the concatenated dataset consisting of the 14 protein coding mt genes to the known phylogenies based on nuclear genes [25-30]. These analyses also allowed us to take into account the evolutionary histories of the individual mt genes or groups of genes. The latter is an important consideration for the GFC because its evolution has been suggested to involve hybridization among ancestral lineages [42], the effects of which are often seen in mitochondrial genomes [43,44].

## Results

### Mitochondrial genome size, organization and gene content

The mt genomes of *F. circinatum* [GenBank accession number JX910419], *F. verticillioides*[22], and *F. fujikuroi* [GenBank accession number JX910420] represent circular molecules with sizes of respectively 67 106, 53 753 and 46 927 base pairs (bp), whereas those of *F. graminearum, F. oxysporum* and *F. solani* are 95 676, 34 476 bp and 62 978 bp, respectively [19,22]. The average GC content of the three GFC genomes were 31.4%, 32.6% and 32.4%, respectively, which fall within the range of what was found for the other *Fusarium* mt genomes [22]. Half (50.5%) of the *F. circinatum* mt genome comprised of protein coding sequences, which is comparable to the 57.6% found in the *F. oxysporum* mt genome. In contrast, only 34.5% of the *F. verticillioides* mt genome, 34% of the *F. fujikuroi* mt genome, 30.8% of the *F. solani* mt genome and 20.9% of the *F. graminearum* mt genome accounted for protein coding genes.

**Figure 1:**
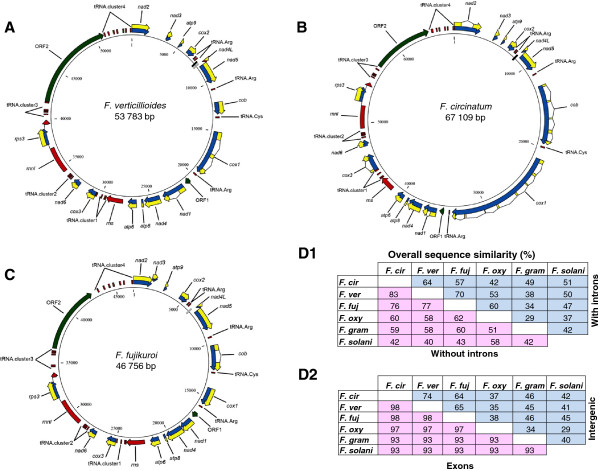
**Maps and identity metrics for the three *Fusarium *mt genomes examined. A**-**C**: Annotated maps for the mt genomes of *F. verticillioides* strain NRRL 29056 (53 783 bp), *F. circinatum* strain MRC 7870 (67 106 bp)*,* and *F. fujikuroi* strain IMI 58289 (46 927 bp). All three genomes encode the 14 protein coding genes of the oxidative phosphorylation pathway (blue = entire gene; yellow = coding sequence), two rRNA (red), 27 tRNA (red) and 2 unique ORFs (green) in identical gene order, transcribed from the same strand. tRNA cluster 1 (*F. verticillioides*) = tRNA.Tyr, tRNA.Asp, tRNA.Ser, tRNA.Asn; tRNA cluster 1 (*F. circinatum, F. fujikuroi*) = tRNA.Tyr, tRNA.Asp, tRNA.Ser, tRNA.Ser; tRNA cluster 2 (*F. verticillioides, F. circinatum, F. fujikuroi*) = tRNA.Val, tRNA.Ile, tRNA.Ser, tRNA.Trp, tRNA.Pro; tRNA cluster 3 (*F. verticillioides, F. circinatum*) = tRNA.Thr, tRNA.Glu, tRNA.Met, tRNA.Met, tRNA.Gly, tRNA.Leu; tRNA cluster 3 (*F. fujikuroi*) = tRNA.Met, tRNA.Arg, tRNA.Thr, tRNA.Glu, tRNA.Met, tRNA.Met; tRNA cluster 4 (*F.verticillioides*, *F. circinatum*, *F. fujikuroi*) = tRNA.Ala, tRNA.Phe, tRNA.Lys, tRNA.Leu, tRNA.Gln, tRNA.His, tRNA.Met. Repeats shared between the mt genomes are mapped as follows: black = repeat motif shared between *F. circinatum* and *F. verticillioides*; grey = repeat motif shared between *F. verticillioides* and *F. fujikuroi*. **D1**: Overall sequence identity (above the diagonal) and overall sequence identity excluding intron regions (below the diagonal). **D2**: Sequence identity of exons (above the diagonal) and overall sequence identity of the intergenic regions (below the diagonal). Overall sequence identity includes the sequence data for *F.verticillioides* (*F. ver*), *F. circinatum* (*F. cir*) and *F. fujikuroi* (*F. fuj*), as well as the other *Fusarium* species with available mt sequences. These are *F. oxysporum* (*F. oxy*) (AY945289) and *F. graminearum* (*F. gram*) (DQ364632) and *F. solani* (*F. solani*) (JN041209).

The *F. circinatum* and *F. fujikuroi* mt genomes like all other *Fusarium* mt genomes, harbour 14 protein coding genes involved in oxidative phosphorylation. These include the genes encoding three cytochrome c oxidase subunits (*cox*1, *cox*2 and *cox*3), cytochrome b (*cob*), three adenosine triphosphate (ATP) synthase subunits (*atp*6, *atp*8 and*atp*9), and seven nicotinamide adenine dinucleotide (NADH) dehydrogenase subunits (*nad*1, *nad*2, *nad*3, *nad*4, *nad*5, *nad*6 and *nad*4L) (Figure 1). Again, the two genomes also harboured genes encoding the large (*rnl*) and small (*rns*) subunit ribosomal RNAs, one small subunit ribosomal protein 3 (*rps*3; located within the Group I intron of *rnl*), and 27 tRNA genes (see below; Figure 1). In these mt genomes, all of the predicted protein coding genes were located in identical order and were encoded on the same strand, which is similar to what was shown for *F. verticillioides*, *F. oxysporum, F. graminearum* and *F. solani*[19,22].

Apart from the genes commonly present in the mt genomes of most fungi [5], a number of additional open reading frames (ORFs) were also identified in this study. However, the majority of these ORFs did not match the criteria for being putatively functional [14]. This is because they had a translation initiation codon that was different to the known mt genes, were smaller than the smallest known mt gene (i.e., *atp*8*,* 52 amino acids) and showed no significant similarity to those in the non-redundant protein database of the National Center for Biotechnology Information (NCBI, http://www.ncbi.nlm.nih.gov/) (data not shown).Only two of the unique putative ORFs matched the criteria set by Formighieri *et al.*[14] and potentially represent genes with functional products. These two ORFs were present in all the GFC genomes examined and at similar positions, i.e., upstream of *nad*1 (ORF1) and within the large tRNA cluster (ORF2) (Figure 1). ORF2 was also present in the mt genome of *F. verticillioides, F. graminearum* and *F. solani*, whereas ORF1was present in all the *Fusarium* mt genomes including *F. oxysporum* in similar locations compared to *F. circinatum* and *F. fujikuroi*[22].

**Figure 2:**
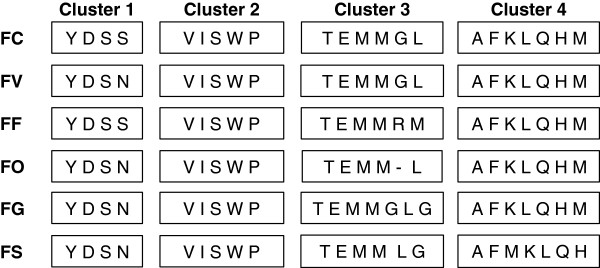
**Comparison of the tRNA gene clusters (see Figure**1**A-C) of *Fusarium circinatum *(FC) (JX910419), *F. verticillioides *(FV) (JN041210), *F. fujikuroi *(FF) (JX910420), *F. oxysporum *(FO) (AY945289), *F. graminearum *(FG) (DQ364632) and *F. solani *(FS) (JN041209).** The tRNA genes in each cluster are indicated by using the standard one-letter abbreviations for the specific amino acids they carry.

The majority of the tRNA genes were located within four tRNA clusters, which were respectively downstream of *rns* (cluster 1 with four tRNA genes), *nad*6 (cluster 2 with five tRNA genes), *rnl* (cluster 3 with six tRNA genes) and ORF2 (cluster 4 with seven tRNA genes) (Figures 1 and 2). The clustering is in agreement to what is known for Sordariomycete mt genomes [20], although the unique ORF2 divided the large tRNA cluster into two smaller clusters. In addition, *F. circinatum* and *F. fujikuroi* also code for five single tRNA genes in identical gene order and at comparable positions to those identified in *F. verticillioides*[22] (Figure 1). Although the mt genomes of *F. oxysporum, F. graminearum* and *F. solani* also code for single tRNA genes at comparable positions to the GFC genomes [22], there are minor differences. These were located upstream of *cox*2 where *F. oxysporum* and *F. solani* encode a single tRNA for Arginine and *F. graminearum* encode an additional tRNA for Tyrosine. Also, all of the examined mt genomes encoded a tRNA gene for Arginine upstream of *nad*5, except for *F. graminearum* and *F. solani* that lacked tRNA genes at this position. Overall, the mt genome of *F. verticillioides, F. graminearum* and *F.solani* encoded tRNA genes corresponding to all 20 amino acids. The mt genome of *F. circinatum* lacked a gene encoding an Asparagine tRNA, while both *F. fujikuroi* and *F.oxysporum* lacked a tRNA gene for Glycine (Figure 1 and Additional file 1: Table S1). Translation of certain mt genes is thus dependent on tRNAs that are produced and imported from the cytosol [2,5] or tRNA genes that are post-transcriptionally modified [45].

Among the three GFC genomes, clusters 2 and 4 had the same tRNA gene order and content, while cluster 1 of *F. verticillioides* differed from that of the other two species in that one of its genes encoded a tRNA for Aspartate rather than Serine (Figure 2). For cluster 3, the *F. circinatum* and *F. verticillioides* genomes were identical in tRNA gene order and content (Figure 2). However, the *F. fujikuroi* cluster 3 encoded the tRNA for Arginine and an additional tRNA for Methionine, as opposed to *F. circinatum* and *F. verticillioides* where the tRNA in the same locus had the Glycine and Leucine tRNAs (Figure 2). Overall, however, the tRNA gene clusters of the GFC species are nearly identical to those of the other *Fusarium* species (Figure 2), which is consistent with the tRNA gene cluster conservation reported for Sordariomycetes [20]*.* The minor tRNA differences observed for the species within GFC could potentially be differences linked to the three Clades of GFC or it could be unique for each species, the elucidation of which depends on the availability of mt genome sequence information for more species in the complex.

The overall percentage nucleotide sequence identity among the three GFC mt genomes ranged from 57 to 70% (Figure 1D). When the other species of *Fusarium* were included in the analyses this overall nucleotide identity range were 29 to 70%. This overall sequence identity increased considerably (76-83% for GFC and 42%-83% including all *Fusarium* species) after exclusion of the introns from the pairwise comparisons. For example, introns accounted for respectively 56.8%, 21%, 7.8%, 6%, 77 % and 58.3% of the *F. circinatum*, *F. verticillioides*, *F. fujikuroi*, *F. oxysporum*, *F. graminearum* and *F. solani* nucleotide identity in protein coding regions. The identity among the genomes was even more evident when only the exons of the protein coding genes were considered with all comparisons yielding nucleotide identity values >93%. This identity was also evident from the results of comparative BLAST analyses (Additional file 1: Figure S1). In terms of the intergenic regions, the nucleotide identity (calculated from the averages of individual alignments) shared among the GFC genomes ranged from 63% to 74%, whereas inclusion of the other *Fusarium* species yielded nucleotide identity values from 29 to 74% (Figure 1D). These values are thus consistent with the expectation that the mt sequences for species in the GFC are more similar to one another than to those for species outside the complex. The majority of the size and sequence differences observed in the mt genomes of the GFC species (and the other *Fusarium* species) were mostly linked to the sequence heterogeneity of the intergenic regions and the small number of shared introns (see below).

### Codon usage

The protein coding genes and the two unique ORFs for the sequenced mt genomes all started with the translation initiation codon ATG, whereas the preferred stop codon appears to be TAA, with TAG as an alternative stop codon (Additional file 1: Table S1). The most frequently used codons for all three GFC mt genomes were TTA, ATA and TTT that code for the highly hydrophobic amino acids leucine (leu), isoleucine (ile) and phenylalanine (phe). All three genomes were characterized by missing codons or codons that were under-represented (less than 10). For example, codons CTC (leu), AGG (arg) and CGG (arg) were missing from *F. circinatum* while codons TGC (cys), GGC (gly), AAG (lys), CAG (gln), CGA (arg), CGT (arg), CGC (arg), TCG (ser), ACG (thr), ACC (thr), GTC (val) and TGG (trp) were under-represented in this genome (Additional file 1: Table S1). Missing or under-represented codons were mostly those with a third position G or C, supporting the third codon position bias towards A and T that were generally observed in the data (Additional file 1: Table S1). For the known mt genes, we observed a similar codon bias within and between genomes and a similar codon bias was observed for the other *Fusarium* mt genomes described previously [22]. However, the two unique ORFs differed from the known genes with regards to the tryptophan codon TGG, which is under-represented in the other genes, but occurred relative to the alternative codon (TGA) for this amino acid, in a 1:1 ratio in *F. circinatum* and *F. fujikuroi.* These data thus suggest that the unique ORFs have codon biases different from those of the 14 known protein coding genes, in agreement to what was observed for the other *Fusarium* mt genomes [22].

### Intergenic repeat elements

Motifs consisting of 15 nucleotides or more and occurring more than once were identified in all three mt genomes (Additional file 1: Table S2). The *F. verticillioides* sequence harboured 39 repeat motifs of which nine represented direct repeats (repeat units in the same orientation within the same intergenic region), 27 were dispersed direct repeats (direct repeat units dispersed between genes), one was a direct and a dispersed repeat (direct repeat units in the same intergenic region as well as dispersed between genes) and 16 direct inverted repeats (repeat units in the same intergenic region with the second repeat in reverse orientation). The *F. circinatum* mt genome contained 33 intergenic repeat elements of which five were direct repeats, 15 dispersed direct, four direct and dispersed repeat and nine were direct inverted repeats. The intergenic regions of *F. fujikuroi* contained 21 repeats of which nine were direct, six were dispersed direct and 9 were direct inverted repeats. The copy number of the majority of these motifs were two, although some were as high as 9. For example, within *F. circinatum* one dispersed repeat was repeated five times, while two direct and dispersed repeats were repeated five and six times, respectively. Within the intergenic regions of *F. verticillioides* two dispersed repeats were repeated four and seven times and one direct and dispersed repeat were repeated nine times. *F. fujikuroi* had only one direct repeat that was repeated four times. Repeat motifs were also distributed randomly throughout the mt genomes and were therefore not limited to specific intergenic regions.

Repeat motifs were unique for each species. There were two exceptions where *F. circinatum* and *F. verticillioides* shared one repeat and *F. verticillioides* and *F. fujikuroi* shared another (Figure 1 and Additional file 1: Table S2). The repeat motifs shared between these pairs were located within the intergenic region between the *cox2* and *nad4L* genes. However, the repeats identified in this study were unique to the GFC, because none of the identified motifs were found within the genomes of *F. oxysporum*, *F. solani* or *F. graminearum*.

### Introns

**Figure 3:**
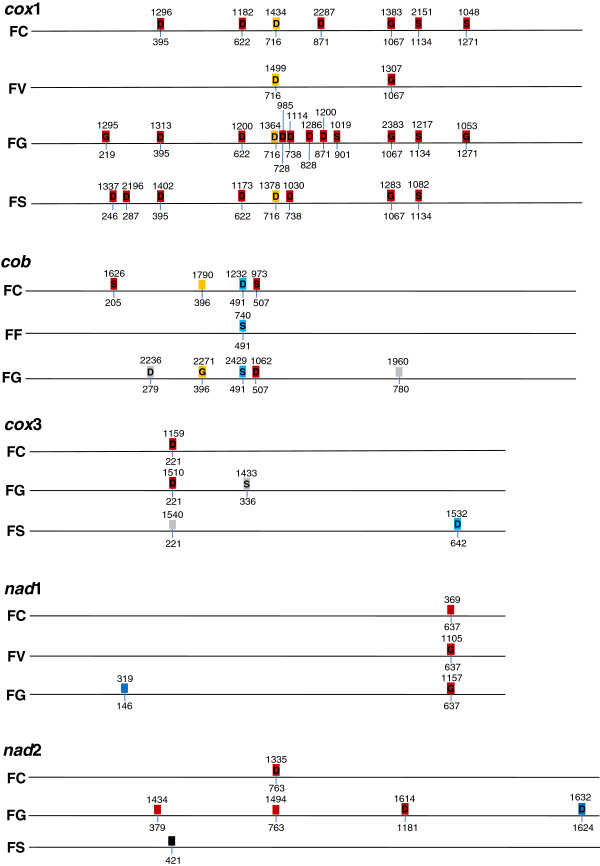
**Intron insertion position, intron type, and endonuclease within the *cox*1*, cox*3*, cob, nad*1*, nad*2 genes of *Fusarium circinatum *(FC)(JX910419), *F. verticillioides *(FV)(JN041210)*, F. fujikuroi *(FF) (JX910420)*, F. oxysporum*(FO) (AY945289)**, ***F. graminearum *(FG)(DQ364632) and *F. solani *(FS) (JN041209).** Each block colour represents a different group I intron: blue = group1A, red = group1B, grey = group1C, yellow = group1D and black = GroupII. The G inside the intron indicates the presence of a GIY-YIG endonuclease, while S indicate a single LAGLIDADG and D a double LAGLIDADG. Intron insertion positions are indicated below the intron and intron sizes are indicated above the intron.

Consistent with what is known for fungal mt genomes, none of the predicted tRNA genes in the *F. fujikuroi* and *F. circinatum* mt genomes contained introns. Within these mt genomes 5 [22], 2 and 15 introns were, respectively, identified. The *rnl* gene harboured a single intron in all three GFC genomes. The *F. circinatum* mt genome contained 14 additional introns within the *nad*2, *cob*, *cox*1, *nad*1, and *cox*3 genes, the *cob* gene of *F. fujikuroi* also contained an intron (Figure 3 and Additional file 1: Table S3), while the *nad*1, *nad2* and *cox*1 genes of *F. verticillioides* contained introns. This variation in intron density was also observed in the mt genomes for species outside the GFC, where *F. oxysporum* contained 2 introns, *F. graminearum* contained 34 and *F. solani* 15 [22]. To evaluate the level of variation in intron content and position that might be expected among isolates, we used primers flanking introns identified within the *cox*1 gene to determine the presence of these introns in a set of eight additional isolates each of *F. circinatum* and *F. verticillioies*. The results (not shown) of the PCR reactions with these primers showed that some of the introns were absent from three of the examined *F. circinatum* isolates, while two of the *F. verticillioides* isolates harboured additional introns.

The only intron that was shared between all three mt genomes (i.e., the one *rnl*) is a group I intron which is inserted after the central loop of domain V [20]. This intron also harbours the gene encoding the small subunit ribosomal protein *rps3.*RNA secondary structure-based BLAST comparisons of the introns identified within the protein coding genes suggest that these also represent group I introns. Based on their higher-level structures, these self-splicing elements were further classified as group IA, group IB, group IC or group ID (Additional file 1: Table S3). The identified introns ranged in length from 357 bp to 2287 bp (Additional file 1: Table S3), with the larger introns harbouring homing endonuclease encoding HEGs of the LAGLIDADG and/or GIY-YIG family. Two of the *F. circinatum* introns (one in *cox1* and the other in *cob*) were also “biorfic” as they contained two HEGs. These ORFs were either in frame with the exon, or were inserted within the P1 or P8 regions of the intron. No HEG was identified outside intron regions within intergenic regions.

In order to determine if the introns encoded different LAGLIDADG and/or GIY-YIG homing endonuclease family members [46], their functional domains were compared phylogenetically. The resulting trees separated the HEGs into various small groups (Additional file 1: Figures S2 and S3). HEGs identified from the same species or the same gene within a species from both phylogenies did not group together. In fact, phylogenetic groups mostly comprised of HEGs from all the *Fusarium* species identified within similar intron positions. For example, the *F. circinatum*, *F. verticillioides*, *F. solani* and *F. graminearum cox1* GIY-YIGs in the intron at position 1067 bp (based on the nucleotide position of the exon) grouped together, which was also true for the LAGLIDADGs in the intron at position 716 bp of this gene (Additional file 1: Figures S2 and S3).

Despite the overall variation with regards to number, size and type of HEG among the genomes, intron positions appeared to be conserved (Figure 3 and Additional file 1: Table S3). For example, the position of the two introns within the *cox1* gene of *F. verticillioides* corresponded to the two insertion positions in *cox1* of *F. circinatum*, while the position of all of the *F. circinatum cox1* introns were similar to those of seven introns in the *F. graminearum cox1* (Figure 3). Introns inserted at shared positions were also mostly similar in terms of length, intron subgroup, HEG position, HEG length, and HEG sequence (Additional file 1: Table S3, Figures S2 and S3). Where minor differences were detected among introns sharing similar positions, these mostly occurred when the mt genome of *F. graminearum* or *F. solani* was compared to those of the three species in GFC. For example, the introns in position 1067 of the *cox1* gene in *F. circinatum* (intron 5), *F. verticillioides* (intron 2), *F. solani* (intron 7) and *F. graminearum* (intron 10) belong to the same subgroup (1B) and contain the same HEG, although in *F. graminearum,* this intron was larger because it contained an additional HEG (Additional file 1: Table S3; Figure 3). If we compare the intron insertion positions of the *cox*1 gene of *F. solani* to *F. graminearum* and the GFC species, two unique insertion positions are present within *F. solani* although it contained less introns compared to *F. graminearum.*

### Comparison of phylogenies inferred from protein coding genes

**Figure 4:**
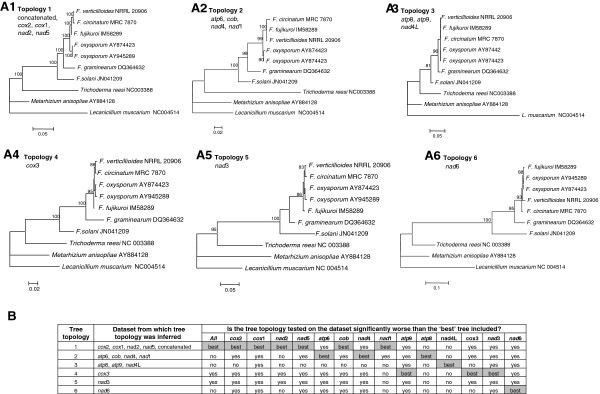
**Comparison of the tree topologies inferred from the various single and concatenated mitochondrial gene datasets. A**. Maximum likelihood phylogenies for *Fusarium* based on mt protein coding nucleotide sequences. The corresponding sequences for three species *Trichoderma reesi*, *Metarhizium anisopliae* and *Lecanicillium muscarium* in the *Hypocreales* were used for outgroup purposes. Bootstrap values (>85%), based on 1000 replications are indicated at the internodes. 1: Phylogeny based on the concatenated data set for known mitochondrial genes. A similar topology (referred to as topology 1) was inferred from the single gene datasets for *cox*2*, cox*3, *nad*2 and *nad*5*.*2: Phylogeny based on the *atp*6 dataset with similar topologies (topology 2) inferred from the *cob, nad*4 and *nad*1single gene datasets*.* 3: Phylogeny based on the *atp*8 dataset, with similar topologies (topology 3) inferred from the *atp*9 and *nad*4L single gene datasets*.* 4: Phylogeny (topology 4) inferred from the *cox*1 dataset*.* 5: Phylogeny (topology 5) inferred from the *nad*3 dataset*.* 6: Phylogeny (topology 6) inferred from the *nad*6 dataset*.***B**. Summarized results of Shimodaira-Hasegawa (SH) tests where support for the six tree topologies was tested against the various datasets. For each dataset, the tree receiving the best likelihood score are indicated with “Best”; those topologies that are significantly worse (P < 0.05) than the best tree are indicated with “Yes” and those that are not (P > 0.05) with “No”. See Additional file 1: Table S3 for the full results.

Phylogenetic analyses of the concatenated datasets consisting of the nucleotide sequences for the 14 protein coding sequences grouped *F. circinatum*, *F. verticillioides* and *F. fujikuroi* as close relatives, separate from the other *Fusarium* species included in this study (Figure 4 A1)(nucleotide sequences) (Topology 1). Analysis of the amino acid sequences for this concatenated dataset generated a similar tree (results not shown). According to this phylogeny, the “Asian” Clade emerged first from the common ancestor followed by the divergence of the “African” and “American” Clades, because *F. circinatum* and *F. verticillioides* had a separate sister group relationship with *F. fujikuroi* at their base. Phylogenies constructed from individual nucleotide gene regions however did not always support the groupings recovered from the concatenated dataset (Additional file 1: Figure S4). Of the 14 gene regions tested, only *cox*1, *cox*2, *nad*2 and *nad*5 yielded trees similar to Topology 1 (Figure 4, A1). Phylogenetic analyses of the *atp*6, *cob*, *nad*4 and *nad*1 datasets did not allow resolution of the three GFC species (Figure 4 A2) (Topology 2), while analyses of the *atp*8, *atp*9 and *nad*4L datasets clustered the GFC species together with *F. oxysporum* and *F. graminearum* (Figure 4 A3)(Topology 3). Analyses of the *cox*3, *nad*3 and *nad*6 datasets all generated unique phylogenies (Figure 4 A4, A5, A6)(Topologies 4, 5 and 6) in which the GFC species were polyphyletic, with *F. fujikuroi* grouping away from the other GFC species.

Each of the six topologies recovered in this study (Figure 4 A1-6) were subjected to Shimodaira-Hasegawa (SH) [47] tests to evaluate their level of support by the various individual and concatenated mt gene datasets. These results showed that the six phylogenetic hypotheses are not supported by all the datasets or partitions (Figure 4B, Additional file 1: Table S4). For example, Topology 1could be rejected (P < 0.05) when the *cox*3*, nad*3 and *nad*6 datasets were tested, while the only datasets not resulting in the rejection of Topologies 4, 5 or 6 were respectively *cox*3*, nad*3 and *nad*6 (from which it was inferred). These results thus show that the *cox*3*, nad*3 and *nad*6 sequence data strongly support phylogenies in which the GFC is polyphyletic. The fact that some datasets, especially the *atp*8, *nad*1 and *nad*4L sequences appeared to support multiple topologies probably reflect an overall lack of phylogenetically informative sites in these genes (Additional file 1: Figure S4).

In order to determine if incongruence between individual mt genes and concatenated mt datasets is common or potentially unique to the *Fusarium* species in the GFC, we conducted similar phylogenetic analyses on *Saccharomyces* species for which a known species tree is available [48]. However, the topologies recovered from individual mt genes were generally the same as that of the tree recovered from the concatenated dataset and that was described by Gaillardin *et al*. [48] (Additional file 1: Figure S5). The only exception was *cox*2 which did not confidently group *S. castellii* and *S. servazzi*. None of the single gene datasets for these *Saccharomyces* species thus significantly supported a topology different from the one inferred from the concatenated dataset, which is very different from what was observed for the *Fusarium* species examined in the current study.

## Discussion

Results of this study revealed a high level of similarity among the mt genomes of *F. circinatum*, *F. verticillioides* and *F. fujikuroi*. Many of the shared characters observed in these genomes are also common among the mt genomes of other Sordariomycetes [18-22,41]. This is evident from the overall synteny of the genomes, particularly with respect to gene order conservation of the *nad2-nad3*, the *nad*4L-*nad*5, the *cob-cox1*, the *nad1-nad4*, the *atp8-atp6* genes, *rns-trn-cox*3-*trn* and *nad*6-trn genes.The mt tRNA genes of all Sordariomycetes examined so far are clustered and we also observed the characteristic conservation of gene order. Additional properties typical of the mt genomes of Sordariomycetes include transcription from the same strand, *nad*4L and *nad*5 genes overlapping by one nucleotide and biases towards TAA and ATG in stop and start codons, respectively. Although the mt genomes of Sordariomycetes are generally diverse with regards to intron density and type, these genomes are all characterized by the presence of a group I intron after the central loop of domain V in the *rnl* gene, which harbours an ORF encoding *rps*3. In agreement to what is typically found in other Sordariomycetes, the intergenic regions of *Fusarium* mt genomes were also highly diverse with much of the inter-specific sequence dissimilarity attributable to these regions.

The major tRNA gene cluster identified in the *F. circinatum*, *F. verticillioides* and *F. fujikuroi* mt genomes are interrupted by a unique ORF. The presence of this ORF could be a unique characteristic of all *Fusarium* species as it was recently also found in *F. solani* and *F. graminearum*[22]. This suggests that the ORF could have been acquired by the most recent common ancestor of these species, possibly via a horizontal gene transfer event [49,50]. The current analysis of codon usage also supported the notion that this ORF does not share the same ancestry as the rest of the genes on the mt genomes of these fungi, primarily because its codon usage differed from those of the known protein coding genes [51]. Although this unusual codon bias could indicate that the ORF represents a pseudogene [14], the results of Al-Reedy *et. al.*[22] confirming that this ORF is transcribed and encodes a membrane associated protein, suggest otherwise.

The sizes of the mt genomes sequenced for isolates of *F. circinatum*, *F. verticillioides* and *F. fujikuroi* differed considerably. The mt genome sequence of *F. circinatum* is approximately 13 323 and 19 350 bases larger than those of *F. verticillioides* and *F. fujikuroi*, respectively. These size differences were primarily due to the presence of group I self-splicing introns. This is because the exclusion of intron sequences resulted in mt genomes sizes that did not differ by more than 4100 bp (some of which reflect the presence of unique intergenic repeats). This variation in intron number and size was also observed in the *F. oxysporum*, *F. graminearum* and *F. solani* mt genomes*.* However, PCR analysis of the introns located in the *cox*1 gene of a small set of isolates of *F. circinatum* and *F. verticillioides* indicated that some introns were absent from certain individuals, while other individuals harboured additional introns not present in the sequenced isolates, which suggests that the mt genome sizes reported here are not fixed for each species. Although the involvement of potential PCR artifacts cannot be excluded, our results is consistent with what was shown previously for species within the genus *Leptographuim*[52].

The results of analyses of group I introns suggest ancestry involving both horizontal and vertical acquisition for these elements in the GFC [53-57]. The large diversity of introns in the mt genomes of *F. circinatum*, *F. verticillioides* and *F. fujikuroi* and the other *Fusarium* species outside of this complex [22] indicates that the acquisition of these elements occurred multiple times and independently [53,55,56]. In contrast, the fact that introns at comparable positions in genes appear to be ‘orthologous’, or at least encoding sequences sharing a high degree of similarity, suggests vertical transmission or inheritance from a common ancestor with subsequent loss in specific species and/or lineages [54-56]. The apparent conservation of certain intron positions in mt genes could be that these positions are more favorable for intron insertion (i.e., linked to the internal guide sequence) [58]. Intron insertion position conservation within fungal mt genomes [59] and even across kingdoms [57,58] have also been observed in recent studies.

Phylogenetic comparison of the LAGLIDADG and GIY-YIG domains identified in the HEGs of *F. circinatum*, *F. verticillioides* and *F. fujikuroi* and the other *Fusarium* mt genomes [22] reflected the known diversity of the HEGs encoding them [46]. These genes are believed to have invaded group I introns with little association between intron class and HEG type and/or family [46,60]. The LAGLIDADG and GIY-YIG phylogeny not only reflected the diversity of these HEGs in terms of multiple family members, but potentially also reflects the evolution of these elements as “selfish genes” that have acquired nonsense or frameshift mutations once they became fixed [61,62]. Diversity within the HEGs identified from this study could therefore reflect non-functional HEGs, since the cycle of intron-without-HEG to intron-with–functional-HEG to intron-with-nonfunctional-HEG and ultimately back to intron-without-HEG is ongoing [61,62]. However, to determine whether the phylogenetic diversity observed for the LAGLIDADG and GIY-YIG sequences examined here are due to decay through the acquisition of mutations, future experimental analyses would need to consider the functionality (i.e., mobility) of the HEGs within these mt genomes.

The second major objective of this study was to determine whether mt genes represent suitable DNA markers for tracing the evolutionary history of *Fusarium* species in the GFC. To accomplish this, we firstly compared the concatenated phylogeny inferred from the 14 mt protein coding genes with phylogenies inferred from nuclear genes described previously [25-30]. In the concatenated gene tree, *F. fujikuroi* represented the sister group of a *F. circinatum* + *F. verticillioides* clade, which suggests that the “Asian” Clade is ancestral to the “American” + “African” Clades. Although this grouping is in agreement with some reports [e.g. [28,29], it contradicts previous notions that the “African” Clade is the ancestral group of the GFC. The “African” origins hypothesis for the GFC is based on the fact that the “African” Clade is most species rich and diverse and it also includes most mycotoxin producers [23,25,26]. More importantly, this Clade of the GFC is also the only one that includes chlamydospore-forming species, which is a characteristic thought to be common to *Fusarium* species outside of this complex [23,25,26]. Resolution of the evolutionary history of the GFC thus requires more work involving phylogenetic analyses of multiple additional gene regions and/or phylogenomic approaches using the information from whole nuclear genomes.

Next we compared individual mitochondrial gene tree topologies to the topology of the mitochondrial concatenated dataset. The working hypothesis was that the entire mt genome will reflect or support the same genealogical history [63], because it is thought to represent a single replicative unit that is inherited in a maternal fashion [64]. However, at least six distinct phylogenetic trees were recovered from the various single-gene datasets. Furthermore, three of the single-gene did not allow the recovery of a monophyletic GFC (i.e., *F. circinatum*, *F. verticillioides* and *F. fujikuroi* were not grouped together into a single clade). These conflicts thus suggest that caution should be used when individual mt gene sequences are employed for phylogenetic analyses.

The phylogenetic incongruence among the different mt datasets that was observed in this study could be linked to the life history of the GFC. Previous authors have suggested that the origins of this complex are associated with a hybridization event, which gave rise to multiple non-orthologous copies of one of the internal transcribed spacer regions of the ribosomal RNA genes [42]. If this were true, one would expect the different genes in the mt genomes of the species to be incongruent because of recombination that would have taken place among the genomes [31-33]. In fact, recombination among mt genomes is common when individuals are heteroplasmic due to bi-parental inheritance or parental leakage [43,44], and parental leakage is typical during species hybridization [43]. Although the observed incongruence among the mt gene trees in *F. circinatum*, *F. verticillioides* and *F. fujikuroi* support the notion of an ancient hybridization event, further research is needed to unambiguously resolve the early history of this important complex of fungal species.

## Conclusions

The results of this study revealed similarities among the mt genomes of *F. circinatum*, *F. verticillioides* and *F. fujikuroi* that are linked to gene order and gene orientation conservation, as well as introns at similar insertion positions that appeared to be related. Nevertheless, sequence variation among genomes was mostly a consequence of the sequence variation incorporated in introns and intergenic regions, while genome size was mostly a function of intron density and to some extent the size variation of certain intergenic regions. The incongruent phylogenetic trees recovered from the various single-gene datasets suggest that factors such as biased selection and/or recombination among mt genomes, potentially linked to a hybridization event, preclude these sequences from being used for phylogenetic purposes. Thus, future work should focus on the possible recombination of mitochondria during hybridization and a suspected hybrid origin for the GFC.

## Methods

### Genome sequence and assembly

The mt DNA sequences were extracted from various whole genome sequence libraries. *F. circinatum* strain MRC 7870 was sequenced at the University of Pretoria, FABI, South Africa [65] and *F. fujikuroi* strain IMI58289 was sequenced at the University of Munster in Germany [66]. Following whole genome assembly with Newbler (Newbler, http://www.454.com) for *F. circinatum* and *F. fujikuroi*, contigs containing mt DNA sequences were identified using BLAST comparisons. The latter utilized mt genome information for *F. oxysporum* (F11 and VPRI 19292) [19,41] and *F. graminearum* (PH-1) [22]. Additional contigs with mt sequences were assembled *de novo* with the CLC Genomics Workbench software [version 4.0] (CLC bio, Århus, Denmark) from single reads that were initially identified as being of mt origin following a whole-genome reference assembly to *F. oxysporum* and *F. graminearum*. All contigs were exported to BioEdit for manual assembly using the *F. oxysporum* and *F. graminearum* sequences as references. Finally, primer3 (http://primer3.sourceforge.net/) was used to design primers to amplify regions between non-overlapping contigs (two in *F. fujikuroi* and one in *F. circinatum*). These primers were also used for standard Sanger sequencing to fill the gaps and to complete the mt genomes of these two fungi.

### Genome annotation and analysis

For *F. circinatum* and *F. fujikuroi*, mt genes were identified by similarity searches against the mt genes of *F. oxysporum* and *F. graminearum* using BioEdit and the CLC Genomics Workbench. Open reading frames (ORFs) were identified with ORF Finder using genetic code 4 (Mold, Mitochondria) [67] and BLASTp comparisons. Codon usage was calculated with the online tool at http://www.protocol-online.org. Genes encoding tRNAs were identified with tRNAscan-SE [68], which involved prediction of the secondary structure of RNAs to visualize the tRNA cloverleaf structure with typical 7 nucleotide anti-codon loop, amino acid acceptor stem, D and T loops, and a short variable loop situated between the anti-codon and T loops.

Introns were identified using comparisons of sequence alignments against the known mt genes of *F. oxysporum* and *F. graminearum*. Introns were characterized using RNAweasel (http://megasun.bch.umontreal.ca/RNAweasel) [69]. Homing endonuclease genes (HEGs) within intron regions where identified with ORF Finder (genetic code 4; Mold, Mitochondria) and characterized with InterProScan (http://www.ebi.ac.uk/Tools/pfa/iprscan). To test whether the presence of introns is conserved within species, primers flanking introns identified within the *cox*1 gene were designed and 8 additional isolates of *F. circinatum* and of *F. verticillioies* were screened for the presence or absence of introns.

Overall nucleotide sequence identities of *F. circinatum*, *F. fujikuroi* as well as *F. verticillioides*, *F. oxysporum, F. graminearum* and *F. solani* were determined using the CLC Genomics Workbench. Physical maps and a BLAST comparison, which also included the *F. oxysporum* mt genome, were constructed with CGView Server (http://stothard.afns.ualberta.ca/cgview_server/). Intergenic inverted repeats for each genome were identified with Einverted EMBOSS [70] and intergenic exact repeat elements were identified using REPFIND (http://zlab.bu.edu/repfind) with default settings (P-value of 1×10^-5^ and low complexity filtering). The default parameters excluded one nucleotide and dinucleotide tandem repeats (i.e., short simple repeats) and identified only repeats that are expected to occur by chance on average once in 10 000 bp. Finally, we searched within the intergenic regions of the mt genomes of *F. solani, F. oxysporum* and *F. graminearum* in order to determine whether they share any of the GFC repeat elements.

### Phylogenetic analysis

All phylogenetic analyses were based on maximum likelihood (ML) and were performed with PhyML version 2.4.3 [71]. MAFFT version 5.85 (http://align.bmr.kyushu-u.ac.jp/mafft/online/server/) [72,73] and PRALINE (http://www.ibi.vu.nl/programs/pralinewww) were used to generate nucleotide and amino acid alignments respectively. Best-fit substitution models were determined with jModeltest [74] and ProtTest 2.4 [75] for nucleotide and amino acid data, respectively.

Phylogenetic analysis of the various mt genes also included sequences for other species in the *Hypocreales* for which mt genome sequences are available. These were *F. verticillioides* (JN041210) [22], *F. oxysporum* (AY874423, AY945289) [19,41], *F. graminearum* (DQ364632) [22], *F. solani* (JN041209), *Hypocrea jecorina* (NC003388) [76], *Metarhizium anisopliae* (AY884128) [20] and *Lecanicillium muscarium* (NC004514) [21]. The concatenated nucleotide dataset utilized the GTR model with gamma correction (G) to account for among site rate heterogeneity [77], while the concatenated amino acid dataset utilized the CpREV model [78] with equilibrium amino acid frequencies, G and a proportion of invariable cites (I). The individual mitochondrial nucleotide datasets of *cox1*, *cox2, nad5* and *nad6* utilized the GTR + G. The *cox3* nucleotide dataset also utilized the GTR + I. The TIM1 and TIM2 [77] substitution models with G were used for the *atp6, atp9, cob, nad3, nad4* and the *nad1, nad4L* datasets respectively, while TVM [77] with G and TPM2uf [79] were respectively used for *nad2* and *atp8.* ML bootstrap confidence values were based on a 1000 replications. The likelihood of alternative tree topologies was tested using the Shimodaira-Hasegawa (SH) test in PAUP* version 10b [47].

In order to determine if the single-mt-gene-tree incongruence observed in this study are also observed in other fungal groups, we included additional phylogenetic analysis of the mt sequences of *Saccharomyces* species. For this we focused on *Saccharomyces cerevisiae* (NC_001224), *S. pastorianus* (NC_012145), *S. castellii* (NC_003920) and *S. servazzii*, (NC_004918), using *Candida glabrata* (NC_004691) as outgroup species. These species were recently used for phylogenetic comparisons by Gaillardin *et al.*[48]. For ML analysis, the concatenated dataset, *cob* and *cox*1utilized the TVM + G, while TIM1, GTR and TPM1 [79] all with G was used for *atp*8, *atp*6 and *cox*2, respectively. ML bootstrap confidence values were based on a 1000 replications.

Phylogenetic analyses were used to determine relatedness among the identified HEGs encoded in the introns. These analyses also included sequences for the HEGs identified from *F. oxysporum, F. graminearum*. However, due to the general lack of sequence homology outside the motif characteristic of the endonucleaese families [9], only the conserved domains of these genes were used. For those HEGs that contained more than one conserved domain, each domain was individually compared against the HEGs that contained only one domain. ML trees based on amino acid sequences were constructed as described above, and again utilized the CpREVsubstitution model.

## Competing interests

The authors declare that they have no competing interests.

## Authors’ contributions

All authors participated in the conception and design of the study, the interpretation of findings and drafting of the manuscript. GF performed all of the *in silico* and laboratory procedures. All authors have read and approved the final manuscript.

## Supplementary Material

Additional file 1: Table S1 Mitochondrial amino acid codon usage and tRNA anti-codon sequences for *Fusarium circinatum, F. verticillioides* and *F. fujikuroi*. **Table S2.** Inverted, direct and dispersed repeats identified in the mitochondrial genomes of *Fusarium circinatum*, *F. verticillioides* and *F. fujikuroi.***Table S3.** Intron distribution, type, size, and endonuclease of *F. circinatum*, *F. verticillioides, F. fujikuroi, F. oxysporum*, *F. graminearum and F. solani*. **Table S4.** Comparison of the alternative trees using the SH test. **Figure S1.** Physical map and BLAST comparison of the mt genomes of *F. circinatum* against *F. oxysporum*, *F. verticillioides* and *F. fujikuroi*. **Figure S2.** Midpoint rooted maximum likelihood phylogenetic tree of the amino acid LAGLIDADG endonuclease domains identified within intron regions of *F. circinatum, F. verticillioides*, *F. fujikuroi*, *F. oxysporum, F. graminearum* and *F. solani.***Figure S3.** Midpoint rooted maximum likelihood phylogenetic tree of the amino acid GIY-YIG endonuclease domains identified within intron regions of *F. circinatum, F. verticillioides, F. fujikuroi, F. oxysporum*, *F. graminearum* and *F. solani*. **Figure S4.** Maximum likelihood phylogenies for *Fusarium* based on mitochondrial protein-coding nucleotide sequences. **Figure S5.** Maximum likelihood phylogenies for *Saccharomyces* species based on mitochondrial protein-coding nucleotide sequences. Click here for file
