# Supplementary material for: Evidence for inter-specific recombination among the mitochondrial genomes of Fusarium species in the Gibberella fujikuroi complex
Source: BMC Genomics. 2013 Sep 8;14:605. doi: 10.1186/1471-2164-14-605 (PMC3847072; doi:10.1186/1471-2164-14-605)
Supplement: Additional file 1: Table S1 — Mitochondrial amino acid codon usage and tRNA anti-codon sequences for Fusarium circinatum, F. verticillioides and F. fujikuroi. Table S2. Inverted, direct and dispersed repeats identified in the mitochondrial genomes of Fusarium circinatum, F. verticillioides and F. fujikuroi.Table S3. Intron distribution, type, size, and endonuclease of F. circinatum, F. verticillioides, F. fujikuroi, F. oxysporum, F. graminearum and F. solani. Table S4. Comparison of the alternative trees using the SH test. Figure S1. Physical map and BLAST comparison of the mt genomes of F. circinatum against F. oxysporum, F. verticillioides and F. fujikuroi. Figure S2. Midpoint rooted maximum likelihood phylogenetic tree of the amino acid LAGLIDADG endonuclease domains identified within intron regions of F. circinatum, F. verticillioides, F. fujikuroi, F. oxysporum, F. graminearum and F. solani.Figure S3. Midpoint rooted maximum likelihood phylogenetic tree of the amino acid GIY-YIG endonuclease domains identified within intron regions of F. circinatum, F. verticillioides, F. fujikuroi, F. oxysporum, F. graminearum and F. solani. Figure S4. Maximum likelihood phylogenies for Fusarium based on mitochondrial protein-coding nucleotide sequences. Figure S5. Maximum likelihood phylogenies for Saccharomyces species based on mitochondrial protein-coding nucleotide sequences. [file 1471-2164-14-605-S1.docx]

**Evidence for inter-specific recombination among the mitochondrial genomes of *Fusarium* species in the *Gibberella fujikuroi* complex.**

Gerda Fourie^1^, Nicolaas A. van der Merwe^2^, Brenda D.Wingfield^2^, Mesfin Bogale^2^, Bettina Tudzynski^3^, Michael J Wingfield^1^, Emma T Steenkamp^1^.

Departments of Microbiology and Plant Pathology^1^ and Genetics^2^, Forestry and Agricultural Biotechnology Institute (FABI), University of Pretoria, Pretoria, South Africa. University of Munster, Germany^3^.

*gerda1.fourie@fabi.up.ac.za.

**SUPPLEMENTARY MATERIAL**

**Table S1.** Mitochondrial amino acid codon usage and tRNA anti-codon sequences for *Fusarium circinatum, F. verticillioides* and *F. fujikuroi*.

**Table S2.** Inverted, direct and dispersed repeats identified in the mitochondrial genomes of *Fusarium circinatum*, *F. verticillioides* and *F. fujikuroi.*

**Table S3**. Intron distribution, type, size, and endonuclease of *F. circinatum*, *F. verticillioides, F. fujikuroi, F. oxysporum*, *F. graminearum and F. solani*.

**Table S4.** Comparison of the alternative trees using the SH test.

**Figure S1.** Physical map and BLAST comparison of the mt genomes of *F. circinatum* against *F. oxysporum*, *F. verticillioides* and *F. fujikuroi*.

**Figure S2.** Midpoint rooted maximum likelihood phylogenetic tree of the amino acid LAGLIDADG endonuclease domains identified within intron regions of *F. circinatum, F. verticillioides*, *F. fujikuroi*, *F. oxysporum,* *F. graminearum* and *F. solani.*

**Figure S3.** Midpoint rooted maximum likelihood phylogenetic tree of the amino acid GIY-YIG endonuclease domains identified within intron regions of *F. circinatum, F. verticillioides, F. fujikuroi, F. oxysporum*, *F. graminearum* and *F. solani*.

**Figure S4.** Maximum likelihood phylogenies for *Fusarium* based on mitochondrial protein-coding nucleotide sequences.

**Figure S5.** Maximum likelihood phylogenies for *Saccharomyces* species based on mitochondrial protein-coding nucleotide sequences.

**Table S1.** Mitochondrial amino acid codon usage and tRNA anti-codon sequences for *Fusarium circinatum, F. verticillioides*^a^ and *F. fujikuroi* ^b^.

| **Amino acid** | **Codon** | **Codon usage ^c^**  **(*F. circinatum* / *F. verticillioides* /  *F. fujikuroi*)** | **Anti-codon ^d^**  **(*F. circinatum* / *F. verticillioides* /  *F. fujikuroi*)** |
| --- | --- | --- | --- |
| Alanine | GC*G* | *14* / *15* / *12* | TGC / TGC / TGC |
|  | GCA | 81 / 81 / 72 |  |
|  | GCT | 127 / 122 / 111 |  |
|  | GCC | 29 / 34 / 33 |  |
|  |  |  |  |
| Arginine | AG*G* | *0* / *0* / *15* | ACG, TCG, TCT / ACG, TCG, TCT / ACG, |
|  | AGA | 75 / 75 / 72 | TCG, TCT, CCT |
|  | CG*G* | *0* / *0* / *0* |  |
|  | CG*A* | *1* / *1* / *5* |  |
|  | CG*T* | *6* / *8* / *12* |  |
|  | CG*C* | *2* / *0* / *1* |  |
|  |  |  |  |
| Asparagine | AAT | 178 / 180 / 169 | GTC / GTC /GTC |
|  | AAC | 39 / 36 / 40 |  |
|  |  |  |  |
| Aspartic acid | GAT | 87 / 86 / 79 | GTC / GTC / GTC |
|  | GA*C* | 16 / 19 / 19 |  |
|  |  |  |  |
| Cysteine | TGT | 21 / 22 / 31 | GCA / GCA / GCA |
|  | TG*C* | *6* / *6* / *15* |  |
|  |  |  |  |
| Glutamic acid | GAG | 14 / 15 / 14 | TTC / TTC / TTC |
|  | GAA | 106 / 105 / 107 |  |
|  |  |  |  |
| Glutamine | CA*G* | *5* / *7* / *8* | TTG / TTG / TTG |
|  | CAA | 81 / 80 / 79 |  |
|  |  |  |  |
| Glycine | GGG | 24 / 25 / 23 | TCC / TCC / - |
|  | GGA | 121 / 123 / 123 |  |
|  | GGT | 144 / 144 / 135 |  |
|  | GG*C* | *2* / *1* / *1* |  |
|  |  |  |  |
| Histidine | CAT | 54 / 54 / 48 | GTG / GTG / GTG |
|  | CAC | 30 / 31 / 30 |  |
|  |  |  |  |
| Isoleucine | ATA | 365 / 365 / 360 | GAT / GAT / GAT |
|  | ATT | 135 / 133 / 154 |  |
|  | ATC | 32 / 33 / 35 |  |
|  |  |  |  |
| Leucine | TTG | 20 / 20 / 24 | TAA, TAG / TAA, TAG / TAG |
|  | TTA | 499 / 496 / 480 |  |
|  | CTG | 11 / 11 / 11 |  |
|  | CTA | 53 / 54 / 62 |  |
|  | CTT | 52 / 50 / 55 |  |
|  | CT*C* | *0*/*1*/*2* |  |
| Lysine | AA*G* | *7* / *7* / 17 | TTT / TTT / TTT |
|  | AAA | 112 / 111 / 107 |  |
|  |  |  |  |
| Methionine | ATG | 129 / 129 / 117 | CAT / CAT, GTT / CAT, GTT |
|  |  |  |  |
|  |  |  |  |
| Phenylalanine | TTT | 236 / 236 / 239 | GAA / GAA / GAA |
|  | TTC | 124 / 124 / 113 |  |
|  |  |  |  |
| Proline | CC*G* | *10* / *8* / *9* | TGG / TGG / TGG |
|  | CCA | 40 / 40 / 34 |  |
|  | CCT | 86 / 91 / 83 |  |
|  | CCC | 12 / 9 / *12* |  |
|  |  |  |  |
| Serine | AGT | 131 / 130 / 126 | GCT, CGA, TGA / GCT, TGA / GCT, TGA |
|  | AG*C* | 24 / 25 / 29 |  |
|  | TCG | *6* / *6* / *4* |  |
|  | TCA | 91 / 91 / 89 |  |
|  | TCT | 110 / 111 / 112 |  |
|  | TC*C* | *10* / *8* / *11* |  |
|  |  |  |  |
| Threonine | AC*G* | *2* / *4* / *0* | TGT / TGT / TGT |
|  | ACA | 111 / 114 / 108 |  |
|  | ACT | 105 / 101 / 106 |  |
|  | AC*C* | *5* / *4* / *10* |  |
|  |  |  |  |
| Tryptophan | TG*G* | *2* / *2* / *9* | TCA / TCA / TCA |
|  | TGA | 59 / 59 / 51 |  |
|  |  |  |  |
| Tyrosine | TAT | 170 / 172 / 172 | GTA / GTA / GTA |
|  | TAC | 49 / 47 / 55 |  |
|  |  |  |  |
| Valine | GTG | 37 / 36 / 31 | TAC / TAC / TAC |
|  | GTA | 160 / 161 / 160 |  |
|  | GTT | 102 / 102 / 102 |  |
|  | GT*C* | *8* / *7* / *14* |  |
|  |  |  |  |
| End | TA*G* | *4* / *3* / *4* |  |
| End | TAA | 10 / 11 / 10 |  |

^a^  In order to compare codon usage between species within GFC, the mitochondrial genome of *F. verticillioides* described by Al-Reedy *et al.*  were also included.

^b^ Codon usage was calculated with the online tool at <http://www.protocol-online.org>. Missing or under-represented codons and corresponding third position G or C are indicated in italics.

^c^ Codon usage are indicated in the order *F. circinatum, F. verticillioides* and *F. fujikuroi*.

^c^ The tRNA anti-codon for each codon are indicated in the order *F. circinatum, F. verticillioides* and *F. fujikuroi*.

**Table S2.** Inverted, direct and dispersed repeats identified in the mitochondrial genomes of *Fusarium circinatum*, *F. verticillioides* and *F. fujikuroi.*

| **Sequence motif *F. circinatum*** | **Nucleotide position** | **Position relative to other genes** | **Type^a^** |
| --- | --- | --- | --- |
| GAGCTTTAGCTTGCG | 3084, 4500, 4538 | between *nad2* and *nad3* | direct |
| ACGAAGTATGCTTGCGCCGGAATCGCGCAAGCTAGAAAAAAATGT | 3111, 6407 | between *nad2* and *nad3* + *atp9* and *cox2* | dispersed |
| TCGCGCAAGCTAGAA | 3133, 6429, 10695 | between *nad2* and *nad3* + *atp9* and *cox2* + *nad5* and *cob* | dispersed |
| TCTCTAAAAAAAATATATTTTTTTTTATA | 4167, 4346 | between *nad2* and *nad3* | inverted |
| GGGCTGCGCAAGCTAAAGCTC | 4317, 4391, 8174, 33982, 38967 | between *nad2* and *nad3* + *cox2* and *nad4L* + *cox1* and *nad1* + *nad4* and *atp8* | dispersed |
| GCTGCGCAAGCTAAAGCT | 4319, 4393, 8176, 11976 | between *nad2* and *nad3* + *cox2* and *nad4L* + *nad5* and *cob* | direct and dispersed |
| GCGCAAGCTAAAGCT | 4322, 4396, 6536, 8179, 8247, 11979 | between *nad2* and *nad3* + *atp9* and *cox2* + *cox2* and *nad4L* + *nad5* and *cob* | direct and dispersed |
| GAGGGCTGCGCAAGCTAAAGCTCC | 4389, 8172 | between *nad2*and *nad3* + *cox2* and *nad4L* | dispersed |
| AGAGCTTTAGCTTGCGC | 4499, 4537 | between *nad2* and *nad3* | direct |
| GAGCTTCTATTTCATA | 6158, 11611 | between *atp9* and *cox2* + *nad5* and *cob* | dispersed |
| CGCGCAAGCTAAAGC | 6536, 8246 | between *atp9* and *cox2* + *cox2* and *nad4L* | dispersed |
| GCAAGCTAAAGCTCT | 6539, 7819 | between *atp9* and *cox2* + *cox2* and *nad4L* | dispersed |
| AGCTTCTGATTCCCTACGGG | 7636, 7657 | between *cox2* and *nad4L* | direct |
| CGCAAGCTAAAGCTAT | 8247, 8269, 11979 | between *cox2* and *nad4L* + *nad5*and *cob* | direct and dispersed |
| GCTCTTAGCTTTTAGGAGAC | 10 872, 10895 | between *nad5* and *cob* | inverted |
| CACAGTAAGGCGCTAGCTAT | 12557, 12582 | between *nad5* and *cob* | inverted |
| AAAAAAAATGTATTTTTT | 19807, 20049 | between *cob* and *cox1* | inverted |
| CCTACGAGTGACGCTGTGTGCACGTATTATAAT | 34754, 39038 | between *cox1* and *nad1* + *nad4* and *atp8* | dispersed |
| GGCTGCGCAAGCTAAAGCTC | 38968, 40668 | between *nad4* and *atp8* | inverted |
| CTTTAGCTTGCTAAATTATAGCTCGCTATAGCT | 39420, 41139 | between *atp8* and *atp6* | inverted |
| GCGATTTTCTCCGAAAATAC | 43338, 43504 | tRNA cluster upstream of *rns* | dispersed |
| CTCTAACAAAGTGTACGCCTAT | 43426, 46901 | tRNA cluster upstream of *rns +* between *cox3* and *nad6* | dispersed |
| GGAGCTTTAGCTTGCGC | 43405, 48361 | tRNA cluster upstream of *rns + nad6* | dispersed |
| CTAACAACGTGTATTCATAG | 44064, 48479 | tRNA cluster upstream of *rns + nad6* | dispersed |
| CTTGCGCATCGTTCT | 43480, 54162 | tRNA cluster upstream of *rns + rnnl* | dispersed |
| TTTGCTTTTAGCTTTTACCT | 46235, 46416 | between *cox3* and *nad6* | inverted |
| AAGCTCTGTATTTTTTTTATAAA | 46439, 48225 | between *cox3* and *nad6* | inverted |
| TCGTTCTGGCTAGCCAGCC | 46981, 54170, 55347 | between *cox3* and *nad6* + tRNA cluster upstream of *rnnl* | direct and dispersed |
| TATATTTTTTTATATAGC | 48448, 55323 | tRNA cluster upstream of *nad6* + *rnn*l | direct |
| ATAAGGAATTACAGAAAT | 48984, 55097 | tRNA cluster upstream of *nad6 +* *rnnl* | dispersed |
| CGCAAGCTAAAGCTCTCGGAGAAAATCGCGCAAGCT | 48394, 53639 | between *nad6* and *rnnl* | dispersed |
| ATCGTTCTGGCTAGCCAGCC | 54169, 55346 | tRNA cluster upstream of *rnnl* | direct |
| TATAAAAAAAGATATTTTTTTTATAGCCCTCCTC | 65164, 66479 | between ORF2 and *nad2* | inverted |
|  |  |  |  |
| **Sequence motif *F. verticillioides*** | **Nucleotide position** | **Position relative to other genes** | **Type^a^** |
| AGCTTTAGCATGCTG | 1753, 1795 | between *nad2* and *nad3* | direct |
| TGCGCAAGCTAAAGCT | 1997, 4363, 6863, 6894, 7679, 30276, 32013, 38724, 40439 | between *nad2* and *nad3* + *nad3* and *atp9* + *cox2* and *nad4L* + tRNA cluster upstream of *rnnl* | direct and dispersed |
| CGAAGCCGAAAAATATG | 1867, 13493 | between *nad2* and *nad3* + *cob* and *cox1* | dispersed |
| AGCTAGAAAAAAATGTATTTTTTTCTAGCTT | 2009, 5530 | between *nad2* and *nad3* + *atp9* and *cox2* | dispersed |
| GCTAAAGCTCGCTCTC | 3041, 7728 | between *nad2* and *nad3* + *cox2* and *nad4L* | dispersed |
| GAGGGCTAACGAAGTA | 3262, 5748 | between *nad2* and *nad3* + *atp9* and *cox2* | dispersed |
| TTCGGAGAAAATCGC | 4216, 6848 | between *nad3* and *atp9* + *cox2* and *nad4L* | dispersed |
| TCGGAGAAAATCGCGCA | 4217, 6911 | between *nad3* and *atp9* + *cox2* and *nad4L* | dispersed |
| AAATCGCTGCGCAAGCTAAAGCT | 4356, 6856, 6887, 7672 | between *nad3* and *atp9* + *cox2* and *nad4L* | dispersed |
| CTATAAAAAAATATATTTTTTTTTTAACATAGCGAGCTCGCTATGAAATACCGCA | 4382, 5572 | between *nad3* + *atp9* | inverted |
| GCTTCTATTTCGGCG | 4182, 5298 | between *atp9* and *cox2* | direct |
| GCTAGAGCTTGCGCCT | 5349, 11217, 13412, 30228, 30409, 32084, 33310 | between *atp9* and *cox2* + *nad5* and *cob* + *cox1* and tRNA cluster upstream of *nad6* | dispersed |
| GGTGAGCTGGCGCCT | 6834, 7650 | between *atp9* and *cox2* | direct |
| CGGAGAAAATCGCTGCGCAAGCTAAAGCT | 6850, 7666 | between *cox2* and *nad4L* | direct |
| GGAGAAAATCGCTGCGCAAGCTAAAGCT | 6851, 6882, 7667 | between *cox2* and *nad4L* | direct |
| GCAAGCTAAAGCTCT | 6866, 6897, 7121 | between *cox2* and *nad4L* | direct |
| GTAGCGCGAGCTCCCCTCCTAT | 6965, 6990 | between *cox2* and *nad4L* | inverted |
| GATAATTTTGTTTATC | 7257, 7273 | between *cox2* and *nad4L* | direct |
| TTATATATAGCTTGC | 7318, 7455 | between *cox2* and *nad4L* | direct |
| GAGCTTGCGCCTTTCTA | 7557, 13416 | between *cox2* and *nad4L* + between *cob* and *cox1* | dispersed |
| AATTCTTATCCTTATCC | 11329, 11350 | between *nad5* and *cob* | inverted |
| CACAGTAAGGCGCTAGCTAT | 11872, 11897 | between *nad5* and *cob* | inverted |
| TAAAAAAAAAAAGAAAATTTTTTTTTATAGC | 10235, 11175 | between *cob* and *cox1* | inverted |
| GCGCTAGAGCTTGCGCCT | 14797, 19608, 30226, 33308 | between *cob* and *cox1* + *cox1* and *nad1* + tRNA cluster upstream of *rns + nad6* | dispersed |
| GCTTGCTATGAAGGCT | 13964, 20315 | between *cob* and *cox1* + within *nad4* | dispersed |
| TGCTTTAGCTTTTACAGCT | 20011, 20031 | between *cox1* and *nad1* | inverted |
| ACCTACGAGTGACGCTGTGTGCACGTATTATAATTA | 20595, 25748 | between *nad1* and *nad4* + *rns* and *cox3* | dispersed |
| TAGCTTTAGCTTGCTAT | 27540, 27559 | between *atp6* and *rns* | inverted |
| TTTAGCTTGCCTTTAGCTTTTAGCT | 29820, 31588 | between *rns* and *cox3* | inverted |
| AGGTGAGCTGGCGCCTTCGG | 30246, 38694 | tRNA cluster upstream of *rns* + *rnnl* | dispersed |
| TAGCTTTAGCTTGCG | 30087, 37541, 40623 | tRNA cluster upstream of *rns* + *rnnl* | dispersed |
| CTTTAGCTTGCGGAT | 30090, 32043 | tRNA cluster upstream *rns* + *cox3* and *nad6* | dispersed |
| AGGGCTGCGCTAGAGCTT | 33302, 41409 | tRNA cluster upstream of rns + *rnnl* | dispersed |
| ACATTCCTGCACTAGCTAG | 33965, 40194 | tRNA cluster upstream of *rns* and *rnnl* | dispersed |
| AAGGCGCAAGCTCGTG | 32106, 33337 | tRNA cluster upstream of *nad6* | direct |
| GCTATAAAAAAAAAATATATTTTTTTTTTATA | 39258, 40596 | tRNA cluster upstream of *rnnl* | inverted |
| TATGCTTTATAAAAAAAAATATATTTTTTATAGCC | 41068, 41922 | between *rnnl* and ORF2 | inverted |
| TTTATAAAAAAAAATAATTTGTTTATAGG | 50593, 51280 | between ORF2 and *nad2* | inverted |
| GAGCTATATAAAAAAATATATTTTTCTTATAGC | 52069, 52717 | between ORF2 and *nad2* | inverted |
|  |  |  |  |
| **Sequence motif *F. fujikuroi*** | **Nucleotide position** | **Position relative to other genes** | **Type^a^** |
| AAGGCTTTAGCTTAC | 2650, 5636 | between *nad3* and *atp9* + *cox2* and *nad4L* | dispersed |
| TATATTTAGGCGCAAGCTCTAGC | 2357, 5490 | *between nad3 and atp9 + cox2 and* nad4L | dispersed |
| TTTAGCTTACGCAAGC | 2765, 5641 | between *nad3* and *atp9* + *cox2* and *nad4L* | dispersed |
| AGCTTGCACGGGACGGGTAAAAATGTA | 3227, 3442 | between *atp9* and *cox2* | direct |
| GAAAAATCAGCCAAAG | 3982, 13454 | between *atp9* and *cox2* + *cob* and *cox1* | dispersed |
| TTAGTAGTAGCGCGAGC | 5457, 5532 | between *cox2* and *nad4L* | inverted |
| GATAATTTTGTTTATC | 5791, 5807 | between *cox2* and *nad4L* | direct |
| TCAGCATGCTAAAGCT | 6227, 6269 | between *cox2* and *nad4L* | direct |
| TAGAGCTTGCGTAGAGCTTGCGTAGAGCTTG | 8703, 8725, 8747, 8769 | between *nad5* and *cob* | direct |
| GTAGAGCTTGCGTAGAGCTTGC | 8735, 8757 | between *nad5* and *cob* | direct |
| GCTTGCGGCTAAAAAA | 8784, 13111 | between *nad5* and *cob* + *cob* and *cox1* | dispersed |
| CACAGTAAGGCGCTAGCTAT | 9952, 9977 | between *nad5* and *cob* | inverted |
| AAAAAAAAATACATTTTTTTTT | 12753, 13012 | between *cob* and *cox1* | inverted |
| TGCTTTAGCTTTTACAGCT | 16227, 16247 | between *cox1* and ORF1 | inverted |
| AATTTTAAACGTATTAGATTATTCTATGAA | 16546, 20167 | between ORF1 and *nad1* | direct |
| CTATGAAGGCGCAAGCTC | 24804, 26664 | between *cox3* and *nad6* + *nad6* and *rnnl* | dispersed |
| CGAGCTTTAGCTAGCTAGCCGCCGAAAAA | 26665, 27967 | between *cox3* and *nad6* | inverted |
| AGGCTGGCGCAAGCTATGC | 33490, 34926 | between rnnl and ORF2 | inverted |
| AAAAAAAAGTATTTTTTTTT | 35640, 36606 | between rnnl and ORF2 | inverted |
| AAAAAAAATGTTAATTTTTTT | 43563, 43639 | between ORF2 and *nad2* | inverted |
| AAGCATACTTCGTTAGCCCTAAAAGAAAGATATTT | 43874, 44224 | between ORF2 and *nad2* | inverted |
| GCTTGCGCCTAAAAAATATATTTTTTTTA | 44578, 46190 | between ORF2 and *nad2* | inverted |

^a^ Direct repeats are repeat units within the same intergenic region. Dispersed direct repeats are direct repeat units within different intergenic regions. Inverted repeats are repeat units within the same intergenic region however the repeat sequence is in a reverse sequence orientation. Inverted repeats for each genome were identified with Einverted EMBOSS and intergenic exact repeat elements were identified using REPFIND (<http://zlab.bu.edu/repfind>). Repeats in blue are the single repeat motif shared between *F. circinatum* and *F. verticillioides* and the repeat in red are the single repeat motif shared between *F. verticilllioides* and *F. fujikuroi*.

**Table S3**. Intron distribution, type, size, and endonuclease of *F. circinatum*, *F. verticillioides, F. fujikuroi, F. oxysporum*, *F. graminearum* and *F. solani*.

| **Intron** | **Type^a^** | **Insertion position ^b^** | **Size** | **Core structure^c^** | **ORF Type^d^** | **Protein length** | **Domains^e^** |
| --- | --- | --- | --- | --- | --- | --- | --- |
| ***cox*1** |  |  |  |  |  |  |  |
| f.cir.cox1.intron1 | group 1B | 395 | 1296 | 44-1183 | LAGLIDADG^D^ | 383 | 109-205; 260-350 |
| f.cir.cox1.intron2 | group 1B | 622 | 1182 | 18-1078 | LAGLIDADG^D^ | 356 | 82-173; 206-311 |
| f.cir.cox1.intron3 | group 1D | 716 | 1434 | 1214-1332 | LAGLIDADG^D^ | 312 | 68-155; 187-283 |
| f.cir.cox1.intron4^f^ | group 1B | 871 | 2287 | 1287-2238 | LAGLIDADG^D^ | 357 | 96-191; 220-320 |
|  |  |  |  |  | LAGLIDADG^S^ | 378 | 247-344 |
| f.cir.cox1.intron5 | group 1B | 1067 | 1383 | 23-193 | GIY-YIG | 455 | 161-254 |
| f.cir.cox1.intron6 | group 1B | 1134 | 2151 | 53-1116 | LAGLIDADG^S^ | 362 | 96-174 |
| f.cir.cox1.intron7 | group 1B | 1271 | 1048 | 857-1040 | GIY-YIG | 281 | 54-151 |
| f.ver.cox1.intron1 | group 1D | 716 | 1499 | 1279-1397 | LAGLIDADG^D^ | 305 | 68-155; 187-283 |
| f.ver.cox1.intron2 | group 1B | 1067 | 1307 | 23-193 | GIY-YIG | 400 | 135-228 |
| f.gram.cox1.intron1 | group1B | 219 | 1295 | 1185-1254 | GIY-YIG | 335 | 82-163 |
| f.gram.cox1.intron2 | group1B | 395 | 1313 | 216-1203 | LAGLIDADG^D^ | 378 | 108-204; 259-350 |
| f.gram.cox1.intron3 | group 1B | 622 | 1200 | 26-1098 | LAGLIDADG^D^ | 359 | 83-179; 204-315 |
| f.gram.cox1.intron4 | group1D | 716 | 1364 | 1157-1275 | LAGLIDADG^D^ | 307 | 62-152; 184-279 |
| f.gram.cox1.intron5 | group 1B | 728 | 985 | 19-953 | LAGLIDADG^D^ | 313 | 87-165; 193-280 |
| f.gram.cox1.intron6 | group 1B | 738 | 1114 | 18-986 | LAGLIDADG^D^ | 324 | 84-165; 196-295 |
| f.gram.cox1.intron7 | group 1B | 828 | 1286 | 48-262 | LAGLIDADG^D^ | 410 | 121-219; 278-380 |
| f.gram.cox1.intron8 | group 1B | 871 | 1200 | 52-1111 | LAGLIDADG^D^ | 352 | 92-187; 212-312 |
| f.gram.cox1.intron9 | group 1B | 908 | 1019 | 23-99 | LAGLIDADG^S^ | 320 | 190-287 |
| f.gram.cox1.intron10^f^ | group 1B | 1067 | 2383 | 35-205 | GIY-YIG | 440 | 147-230 |
|  |  |  |  |  | GIY-YIG | 73 | 1-62 |
| f.gram.cox1.intron11 | group 1B | 1134 | 1217 | 53-1182 | LAGLIDADG^S^ | 379 | 92-170 |
| f.gram.cox1.intron12 | group 1B | 1271 | 1053 | 859-1039 | GIY-YIG | 277 | 92-170 |
| f.solani.cox1.intron1 | group 1B | 246 | 1337 | 989-1309 | LAGLIDADG^D^ | 313 | 31-119; 150-271 |
| f.solani.cox1.intron2 | group 1B | 287 | 2196 | 1058-1357 | LAGLIDADG^D^ | 302 | 33-126; 153-263 |
| f.solani.cox1.intron3 | group 1B | 395 | 1402 | 1116-1194 | LAGLIDADG^D^ | 289 | 24-118; 173-262 |
| f.solani.cox1.intron4 | group 1B | 622 | 1173 | 18-1069 | LAGLIDADG^D^ | 352 | 82-178; 203-308 |
| f.solani.cox1.intron5 | group 1D | 716 | 1378 | 1158-1276 | LAGLIDADG^D^ | 352 | 65-152;184-280 |
| f.solani.cox1.intron6 | group 1B | 738 | 1030 | 18-956 | LAGLIDADG^D^ | 308 | 80-159; 189-285 |
| f.solani.cox1.intron7 | group 1B | 1067 | 1283 | 26-196 | GIY-YIG | 314 | 127-210 |
| f.solani.cox1.intron8 | group 1B | 1134 | 1082 | 34-1040 | LAGLIDADG^S^ | 342 | 95-170 |
|  |  |  |  |  |  |  |  |
| ***cox*2** |  |  |  |  |  |  |  |
| f.gram.cox2.intron1 | group1C2 | 112 | 1511 | 39-264 | LAGLIDADG^D^ | 298 | 17-115; 174-276 |
| f.gram.cox2.intron2 | group 1B | 230 | 1183 | 924-1148 | GIY-YIG | 294 | 58-142 |
| f.gram.cox2.intron3 | group1C1 | 653 | 1865 | 44-274 | GIY-YIG | 185 | 116-175 |
|  |  |  |  |  |  |  |  |
| ***cox*3** |  |  |  |  |  |  |  |
| f.cir.cox3.intron1 | group 1B | 221 | 1159 | 978-1127 | LAGLIDADG^D^ | 304 | 68-155; 187-283 |
| f.gram.cox3.intron1 | group 1B | 221 | 1510 | 1083-1472 | LAGLIDADG^D^ | 340 | 56-157; 182-318 |
| f.gram.cox3.intron2 | group1C2 | 336 | 1433 | 27-255 | LAGLIDADG^S^ | 352 | 160-259 |
| f.gram.cox3.intron2 | group1C2 | 336 | 1433 | 27-255 | LAGLIDADG^S^ | 352 | 160-259 |
| f.solani.cox3.intron1 | group 1B | 221 | 1540 | 803-991 |  |  |  |
| f.solani.cox3.intron2 | group 1A | 642 | 1532 | 1037-1499 | LAGLIDADG^D^ | 426 | 55-153; 200-297 |
| ***cob*** |  |  |  |  |  |  |  |
| f.cir.cob.intron1 | group 1B | 205 | 1626 | 20-177 | LAGLIDADG^S^ | 320 | 61-171 |
|  |  |  |  |  | LAGLIDADG^S^ | 157 | 59-144 |
| f.cir.cob.intron2 | group 1D | 396 | 1790 | 494-647 | no ORF | no ORF | no ORF |
| f.cir.cob.intron3 | group 1A | 491 | 1232 | 967-1177 | LAGLIDADG^D^ | 295 | 51-138; 189-278 |
| f.cir.cob.intron4^f^ | group 1B | 507 | 973 | 24-182 | LAGLIDADG^S^ | 140 | 45-139 |
|  |  |  |  |  | LAGLIDADG^S^ | 107 | 6-89 |
| f.fuj.cob.intron1 | group1A | 491 | 740 | 475-685 | LAGLIDADG^S^ | 131 | 26-116 |
| f.gram.cob.intron1 | group1C1 | 279 | 2236 | 1778-2009 | LAGLIDADG^D^ | 488 | 98-188; 236-349 |
| f.gram.cob.intron2 | group1D | 396 | 2271 | 1049-1168 | GIY-YIG | 292 | 76-164 |
| f.gram.cob.intron3^f^ | group1A | 491 | 2427 | 2179-2373 | LAGLIDADG^S^ | 267 | 73-162 |
|  |  |  |  |  | LAGLIDADG^S^ | 247 | 60-229 |
| f.gram.cob.intron4 | group 1B | 507 | 1062 | 38-986 | LAGLIDADG^D^ | 319 | 74-170; 206-303 |
| f.gram.cob.intron5 | group1C1 | 780 | 1960 | 56-343 | no ORF | no ORF | no ORF |
|  |  |  |  |  |  |  |  |
| ***nad*1** |  |  |  |  |  |  |  |
| f.cir.nad1.intron1 | group 1B | 637 | 367 | 24-220 | no ORF | no ORF | no ORF |
| f.ver.nad1.intron1 | group 1B | 637 | 1105 | 20-180 | GIY-YIG | 305 | 87-175 |
| f.gram.nad1.intron1 | group1A | 146 | 319 | 64-261 | no ORF | no ORF | no ORF |
| f.gram.nad1.intron2 | group 1B | 637 | 1157 | 33-160 | GIY-YIG | 136 | 9-48 |
|  |  |  |  |  |  |  |  |
| ***nad*2** |  |  |  |  |  |  |  |
| f.cir.nad2.intron1 | group 1C | 763 | 1335 | 44-275 | LAGLIDADG^D^ | 421 | 131-231; 291-389 |
| f.gram.nad2.intron1 | group1C2 | 379 | 1434 | 59-302 | no ORF | no ORF | no ORF |
| f.gram.nad2.intron2 | group1C2 | 763 | 1494 | 49-292 | no ORF | no ORF | no ORF |
| f.gram.nad2.intron3 | group1C2 | 1181 | 1614 | 32-269 | LAGLIDADG^D^ | 418 | 137-235; 298-398 |
| f.gram.nad2.intron4 | group1A | 1624 | 1632 | 1322-1553 | LAGLIDADG^D^ | 349 | 61-157; 219-314 |
| f.solani.nad2.intron1 | group II | 421 | 2362 |  |  |  |  |
|  |  |  |  |  |  |  |  |
| ***nad*3** |  |  |  |  |  |  |  |
| f.gram.nad3.intron1 | group1C2 | 91 | 1450 | 42-285 | LAGLIDADG^D^ | 423 | 139-238; 295-395 |
|  |  |  |  |  |  |  |  |
| ***nad4*L** |  |  |  |  |  |  |  |
| f.gram.nad4L.intron1 | group1C1 | 240 | 1822 | 1341-1770 | LAGLIDADG^D^ | 351 | 58-157; 220-319 |
|  |  |  |  |  |  |  |  |
| ***nad*4** |  |  |  |  |  |  |  |
| f.solani.nad4.intron1 | group 1C2 | 505 | 1397 | 17-295 | LAGLIDADG^D^ | 429 | 139-235; 295-396 |
|  |  |  |  |  |  |  |  |
| ***nad*5** |  |  |  |  |  |  |  |
| f.oxy.nad5.intron1 | group 1B | 718 | 1010 | 22-934 | LAGLIDADG^D^ | 304 | 68-195; 182-280 |
| f.gram.nad5.intron1 | group 1B | 718 | 1019 | 26-941 | LAGLIDADG^D^ | 302 | 65-156; 220-319 |
| f.solani.nad5.intron1 | group 1C2 | 324 | 1348 | 38-272 | LAGLIDADG^D^ | 429 | 139-240; 299-399 |
|  |  |  |  |  |  |  |  |
| ***atp*6** |  |  |  |  |  |  |  |
| f.gram.atp6.intron1 | group 1B | 367 | 1450 | 1127-1414 | LAGLIDADG^D^ | 356 | 67-165; 228-323 |
|  |  |  |  |  |  |  |  |
| ***atp*9** |  |  |  |  |  |  |  |
| f.gram.atp9.intron1 | group1A | 181 | 1088 | 32-206 | GIY-YIG | 276 | 55-138 |
|  |  |  |  |  |  |  |  |

^a^ Intron type determined by RNAweasal (<http://megasun.bch.umontreal.ca/RNAweasel>)[57].

^b^ Nucleotide insertion position with regards to the start of the coding sequence.

^c^ The region that contain the core structure of the introns (P3, P4, P6, P7, P8) identified with RNAweasal.

^d^ ORF type identified with ORF finder (genetic code 4; Mold, Mitochondria) and BLASTp comparison.

^e^ Protein domains characterized with InterProScan (<http://www.ebi.ac.uk/Tools/pfa/iprscan>). LAGLIDADG^D^ contained two domains whereas LAGLIDADG^S^ only one.

^f^ Biorfic intron encodes two OFRs.

**Table S4.** Comparison of the alternative trees using the SH test^a^.

| **Tree^b^** | **ln L** | **Δln L** | ***P* Value^c^** | **Significantly worse?^e^** |
| --- | --- | --- | --- | --- |
|  | **Concatenated** |  |  |  |
| *1* | 23946.77288 | (best) |  | best |
| 2 | 24036.16059 | 89.3877 | 0.015 | no |
| 3 | 24392.70239 | 445.92951 | 0.000 | no |
| 4 | 23985.70301 | 38.93012 | 0.217 | yes |
| 5 | 23985.70301 | 38.93012 | 0.217 | yes |
| 6 | 24069.94940 | 123.17652 | 0.001 | no |
|  | ***cox2*** |  |  |  |
| *1* | 1228.15556 | (best) |  | best |
| 2 | 1239.02323 | 10.86767 | 0.298 | yes |
| 3 | 1276.80028 | 48.64472 | 0.003 | no |
| 4 | 1231.13107 | 2.97551 | 0.697 | yes |
| 5 | 1231.13107 | 2.97551 | 0.697 | yes |
| 6 | 1236.25901 | 8.10345 | 0.403 | yes |
|  | ***cox3*** |  |  |  |
| *1* | 1401.62495 | (best) |  | best |
| 2 | 1408.11666 | 6.49171 | 0.349 | yes |
| 3 | 1417.21305 | 15.5881 | 0.082 | yes |
| 4 | 1401.84707 | 0.22212 | 0.821 | yes |
| 5 | 1401.84707 | 0.22212 | 0.821 | yes |
| 6 | 1406.41134 | 4.78639 | 0.501 | yes |
|  | ***nad2*** |  |  |  |
| *1* | 3148.58325 | (best) |  | best |
| 2 | 3215.37298 | 66.78973 | 0.000 | no |
| 3 | 3234.18315 | 85.5999 | 0.000 | no |
| 4 | 3153.04252 | 4.45927 | 0.564 | yes |
| 5 | 3153.04252 | 4.45927 | 0.564 | yes |
| 6 | 3210.77348 | 62.19023 | 0.000 | no |
|  | ***nad5*** |  |  |  |
| *1* | 3300.29017 | (best) |  | best |
| 2 | 3310.49859 | 10.20842 | 0.373 | yes |
| 3 | 3355.77261 | 55.48244 | 0.005 | no |
| 4 | 3304.15995 | 3.86978 | 0.689 | yes |
| 5 | 3304.15995 | 3.86978 | 0.689 | yes |
| 6 | 3309.07299 | 8.78282 | 0.419 | yes |
|  | ***atp6*** |  |  |  |
| *1* | 1414.31761 | 0 | 0.647 | yes |
| 2 | 1414.31761 | (best) |  | best |
| 3 | 1461.32707 | 47.00946 | 0.007 | no |
| 4 | 1427.75361 | 13.436 | 0.159 | yes |
| 5 | 1427.75361 | 13.436 | 0.159 | yes |
| 6 | 1427.75361 | 13.436 | 0.159 | yes |
|  | ***cob*** |  |  |  |
| *1* | 1950.24117 | (best) |  | best |
| 2 | 1950.24117 | 0 | 0.883 | yes |
| 3 | 1999.88279 | 49.64162 | 0.002 | no |
| 4 | 1950.85218 | 0.61101 | 0.819 | yes |
| 5 | 1950.85218 | 0.61101 | 0.819 | yes |
| 6 | 1951.4114 | 1.17023 | 0.745 | yes |
|  | ***nad4*** |  |  |  |
| *1* | 2426.04132 | 0 | 0.66 | yes |
| 2 | 2426.04132 | (best) |  | best |
| 3 | 2476.34155 | 50.30022 | 0.008 | no |
| 4 | 2435.05980 | 9.01848 | 0.336 | yes |
| 5 | 2435.05980 | 9.01848 | 0.336 | yes |
| 6 | 2435.80133 | 9.76001 | 0.302 | yes |
|  | ***nad1*** |  |  |  |
| *1* | 1945.34168 | (best) |  | best |
| 2 | 1946.36185 | 1.02017 | 0.702 | yes |
| 3 | 2070.26604 | 124.92435 | 0.000 | no |
| 4 | 1981.95746 | 36.61578 | 0.013 | no |
| 5 | 1981.95746 | 36.61578 | 0.013 | no |
| 6 | 1982.77271 | 37.43103 | 0.008 | no |
|  | ***atp8*** |  |  |  |
| *1* | 190.34829 | 0 | 0.000 | no |
| 2 | 190.34829 | (best) |  | best |
| 3 | 190.34829 | 0 | 0.000 | no |
| 4 | 190.34829 | 0 | 0.000 | no |
| 5 | 190.34829 | 0 | 0.000 | no |
| 6 | 190.34829 | 0 | 0.000 | no |
|  | ***atp9*** |  |  |  |
| *1* | 401.32802 | 3.15953 | 0.164 | yes |
| 2 | 401.32802 | 3.15953 | 0.164 | yes |
| 3 | 401.32802 | 3.15953 | 0.164 | yes |
| 4 | 398.16849 | (best) |  | best |
| 5 | 398.16849 | 0 | 0.593 | yes |
| 6 | 401.32802 | 3.15953 | 0.164 | yes |
|  | ***nad4L*** |  |  |  |
| *1* | 448.80268 | 0 | 0.000 | no |
| 2 | 448.80268 | 0 | 0.000 | no |
| 3 | 448.80268 | (best) |  | yes |
| 4 | 448.80268 | 0 | 0.000 | no |
| 5 | 448.80268 | 0 | 0.000 | no |
| 6 | 448.80268 | 0 | 0.000 | no |
|  | ***cox1*** |  |  |  |
| *1* | 3224.81067 | 10.70168 | 0.38 | yes |
| 2 | 3232.70819 | 18.59921 | 0.18 | yes |
| 3 | 3279.47385 | 65.36486 | 0.000 | no |
| 4 | 3214.10898 | (best) |  | best |
| 5 | 3214.10898 | 0 | 0.78 | yes |
| 6 | 3240.97086 | 26.86188 | 0.032 | no |
|  | ***nad3*** |  |  |  |
| *1* | 672.47475 | 0.98611 | 0.643 | yes |
| 2 | 676.02756 | 4.53892 | 0.264 | yes |
| 3 | 680.50691 | 9.01827 | 0.106 | yes |
| 4 | 671.48864 | (best) |  | best |
| 5 | 671.48864 | 0 | 0.792 | yes |
| 6 | 673.52633 | 2.03769 | 0.643 | yes |
|  | ***nad6*** |  |  |  |
| *1* | 1147.84359 | 3.70081 | 0.6 | yes |
| 2 | 1152.62491 | 8.48214 | 0.219 | yes |
| 3 | 1161.45593 | 17.31315 | 0.068 | yes |
| 4 | 1146.60988 | 2.4671 | 0.705 | yes |
| 5 | 1146.60988 | 2.4671 | 0.705 | yes |
| 6 | 1144.14278 | (best) |  | best |

^a^ The Shimodaira-Hasegawa tests were conducted in PAUP [89].

^b^ Tree topologies correspond to those presented in Figure 4. Newic format for the various trees are as follows: Tree 1: (DQ364632,((F.fuj,(F.ver,Fsp34)),(AY945289,AY874423))); Tree 2: (DQ364632,(fsp34,F.ver,f.fuj),(AY945289,AY874423)); Tree 3: (DQ364632,(fsp34,F.ver,f.fuj, AY945289,AY874423)); Tree 4: (DQ364632,(((AY874423,AY945289),(f.ver,fsp34)),F.fuj)); Tree 5: (DQ364632,(f.fuj,((f.ver,fsp34),(AY945289,AY874423)))); Tree 6:(DQ364632,(Fsp34, (F.ver,(F.fuj,(AY945289,AY874423))))). Species and isolates are designated as follows: Fsp34 = *F. circinatum*, F. ver = *F. verticillioides*, F. fuj = *F. fujikuroi*, DQ364632 = *F. graminearum*, AY945289 = *F. oxysporum* isolate F11, AY874423 = *F. oxysporum* isolate VPRI 19292

^c^ P values for the SH tests.

^d^ For each dataset, the tree receiving the best likelihood score are indicated with “Best”; those topologies that are significantly worse (P < 0.05) than the best tree are indicated with “Yes” and those that are not (P > 0.05) with “No”.

**Figure S1.** Physical map and BLAST comparison of the mt genomes of *F. circinatum* against *F. oxysporum*, *F. verticillioides* and *F. fujikuroi*. The coding sequences (CDSs) and tRNA genes predicted in the *F. circinatum* genome are indicated in blue and red, respectively. The GC content of the *F. circinatum* mt genome is indicated in black and was plotted (using a sliding window) as the deviation from the average of 31.4% that was calculated over the entire sequence. The map and BLAST comparison was constructed with CGView Server (<http://stothard.afns.ualberta.ca/cgview_server/>).

**Figure S2.** Midpoint rooted maximum likelihood phylogenetic tree of the amino acid LAGLIDADG endonuclease domains identified within intron regions of *F. circinatum* (*F.cir*), *F. verticillioides* (*F.ver*), *F.fujikuroi* (*F. fuj*)*, F. oxysporum* (*F. oxy*), *F. solani* (*F. sol*) as well as *F. graminearum* (*F. gram*)*.* Bootstrap values (>85%), based on 1000 replications are indicated at the internodes. LAGLIDADG1 and 2 = the first and second conserved domain of the double LAGLIDADG.

**Figure S3.** Midpoint rooted maximum likelihood phylogenetic tree of the amino acid GIY-YIG endonuclease domains identified within intron regions of *F. circinatum* (*F.cir*), *F. verticillioides* (*F.ver*), *F.fujikuroi* (*F. fuj*)*, F. oxysporum* (*F. oxy*), *F. solani* (*F. sol*) as well as *F. graminearum* (*F. gram*)*.* Bootstrap values (>85%), based on 1000 replications are indicated at the internodes.

**Figure S4.** Maximum likelihood phylogenies for *Fusarium* based on mitochondrial protein-coding nucleotide sequences. Species and isolates designated as follows: *F. circinatum* (JX910419), *F. verticillioides* (JN041210), *F. fujikuroi* (JX910420), *F. graminearum* (DQ364632)*F.oxysporum* (AY945289, AY874423), *F. solani* (JN041209)  *Trichoderma reesi* (NC 003388), *Metarhizium anisopliae* (AY884128), *Lecanicillium muscarium* (NC004514).

**Figure S5.** Maximum likelihood phylogenies for *Saccharomyces* species based on mitochondrial protein-coding nucleotide sequences.
